# Primary blood-hosts of mosquitoes are influenced by social and ecological conditions in a complex urban landscape

**DOI:** 10.1186/s13071-018-2779-7

**Published:** 2018-04-10

**Authors:** Heather Goodman, Andrea Egizi, Dina M. Fonseca, Paul T. Leisnham, Shannon L. LaDeau

**Affiliations:** 1 Cary Institute of Ecosystem Studies, Millbrook, NY 12545 USA; 2 Monmouth County Division of Mosquito Control, Tick-Borne Disease Laboratory, New Brunswick, NJ 08901 USA; 3 Center for Vector Biology, Entomology Department, Rutgers University, New Brunswick, NJ 08901 USA; 4 Department of Environmental Science and Technology, University of Maryland, College Park, MD 20742 USA

**Keywords:** Arbovirus, Avian, Blood meal, Human, Mosquito, Rat, Vector

## Abstract

**Background:**

Temperate urban landscapes support persistent and growing populations of *Culex* and *Aedes* mosquito vectors. Large urban mosquito populations can represent a significant risk for transmission of emergent arboviral infection. However, even large mosquito populations are only a risk to the animals they bite. The purpose of this study is to identify and assess spatial patterns of host-use in a temperate urban landscape with heterogeneous socio-economic and ecological conditions.

**Results:**

Mosquito blood meals were collected from neighborhoods categorized along a socio-economic gradient in Baltimore, MD, USA. Blood meal hosts were identified for two *Aedes (Ae. albopictus* and *Ae*. *japonicus*) and three *Culex* (*Cx*. *pipiens*, *Cx*. *restuans* and *Cx*. *salinarius*) species. The brown rat (*Rattus norvegicus*) was the most frequently detected host in both *Aedes* species and *Cx. salinarius*. Human biting was evident in *Aedes* and *Culex* species and the proportion of human blood meals from *Ae. albopictus* varied significantly with neighborhood socio-economic status. *Aedes albopictus* was most likely to feed on human blood hosts (at 50%) in residential blocks categorized as having income above the city median, although there were still more total human bites detected from lower income blocks where *Ae. albopictus* was more abundant. Birds were the most frequently detected *Culex* blood hosts but were absent from all *Aedes* sampled.

**Conclusions:**

This study highlights fine-scale variation in host-use by medically important mosquito vectors and specifically investigates blood meal composition at spatial scales relevant to urban mosquito dispersal and human exposure. Further, the work emphasizes the importance of neighborhood economics and infrastructure management in shaping both the relative abundance of vectors and local blood feeding strategies. The invasive brown rat was an important blood source across vector species and neighborhoods in Baltimore. We show that social and economic conditions can be important predictors of transmission potential in urban landscapes and identify important questions about the role of rodents in supporting urban mosquito populations.

## Background

The establishment of an endemic West Nile virus (WNV) cycle involving *Culex* mosquito vectors across the United States and the global spread of *Aedes* mosquitoes have dramatically changed the landscape of arboviral risk in temperate cities [1–7]. Urban landscapes are a mosaic of land cover, with land-use varying from abandoned and unmanaged to highly managed and engineered sites [8], and where human-influenced resource availability and disturbance can limit establishment of some species while favoring others, including medically important mosquito vectors [9–11]. When vector mosquito species reproduce and develop in residential landscapes, the adult females emerge and initiate blood-seeking behavior in close proximity to human and peridomestic animal hosts. Host-use (and specifically human biting rate) is a well recognized and important parameter in infectious disease models and transmission management strategies [12, 13].

Local transmission of arboviruses depends on female mosquitoes biting an infected host and then surviving long enough to bite again. This can occur *via* sequential human biting (i.e. dengue, Zika viruses) or in the case of WNV, the mosquito must bite the zoonotic (avian) reservoir before feeding from a human [14]. The probability of these specific biting sequences occurring depends on mosquito behavior or innate preferences, relative host availability, and adult mosquito longevity [15]. While studies clearly demonstrate that temperate urban habitat can support population growth of some mosquito species [7, 16–18], there is less consensus regarding the spatial generating mechanisms and consequences of host-use variation across urban landscapes.

Temporal and spatial variation in host-use (defined as the relative proportion of blood meals taken from a given host species in a sampled mosquito population) has been shown to influence the timing and potentially, the intensity of WNV infections transmitted by *Culex* mosquitoes [19–23]. Birds are a predominant blood meal source for both *Cx. restuans* and *Cx. pipiens* across a variety of habitat types and regions [24–26]. *Culex pipiens pipiens* (Linnaeus 1758), which prospers in high-nutrient urban habitat as juveniles [6, 20, 27], is a primary human WNV vector, possibly because it shifts between zoophilic and anthropophilic cycles seasonally [14, 23].

*Aedes albopictus* (Skuse, 1894)*,* more commonly known as the Asian tiger mosquito, has spread globally over the last three decades since its expansion from Southeast Asia to the Americas and is now a predominant nuisance species in many temperate cities [28–31], with clear consequences for arboviral transmission. Autochthonous transmission of chikungunya and dengue viruses by *Ae. albopictus* has been documented in temperate and Mediterranean Europe [32–35] and Japan [36–38]. Studies from across its new geographical range suggest extensive feeding plasticity ranging from exclusive human biting in urban landscapes [39–41] to a more generalist diet including humans, wild and peridomestic mammals, birds and even amphibians/reptiles [39, 40, 42–44]. Research in New Jersey, USA found that although *Ae. albopictus* were predominantly anthropophilic, samples from neighborhoods where pets were more likely to be kept outside had a greater proportion of peridomestic mammalian hosts such as dogs and cats [44]. Unlike *Culex* vectors that may disperse several kilometers [45], *Ae. albopictus* remain close to juvenile habitat and few disperse more than 100 meters [46–49]. In urban neighborhoods where rowhome structures and paved streets can limit vector dispersal [50], non-human blood meal hosts could divert mosquitoes from human biting at fine spatial scales (e.g. zooprophylaxis [51, 52]).

*Aedes japonicus japonicus* (Theobald, 1901) was first detected in the United States in 1998 [53] and is a competent vector for several arboviruses, including WNV, eastern equine encephalitis, and La Crosse virus [54–57]. Like *Ae. albopictus*, this species seems to be an opportunistic mammalian feeder, although there are few studies that have evaluated *Ae. j. japonicus* blood meal hosts in the field. One third of engorged females sampled in New Jersey had taken blood from humans, while over 50% of blood meals were from white-tailed deer [58]. Although it has been reported to bite humans and birds in Japan and in laboratory conditions [59], no avian hosts have been identified in recent field studies. Other mammalian hosts detected in the New Jersey study included horse and Virginia opossum.

In this study we sequenced host DNA collected from *Aedes* and *Culex* mosquitoes collected across socio-economically distinct neighborhoods in Baltimore, MD, USA [29, 60]. Baltimore City is a mosaic of distinct neighborhoods, and socio-economic conditions where housing value, educational attainment, crime rates and even life expectancy can vary significantly across short spatial distances [61–63]. There are an estimated 16,000 standing vacant buildings in Baltimore and this abandoned infrastructure has been demonstrated to be productive habitat for mosquitoes and other pests [29, 60]. Blood meal analyses are critical for elucidating the ecological roles of vertebrate host species in supporting mosquito population growth and pathogen transmission cycles. Identifying the composition of mosquito host species across neighborhoods with differing infrastructure management and residential socio-economic status (SES) conditions can help identify how availability and access to blood meal hosts vary across the urban landscape and influence variation in transmission potential.

## Methods

### Study area

**Fig. 1:**
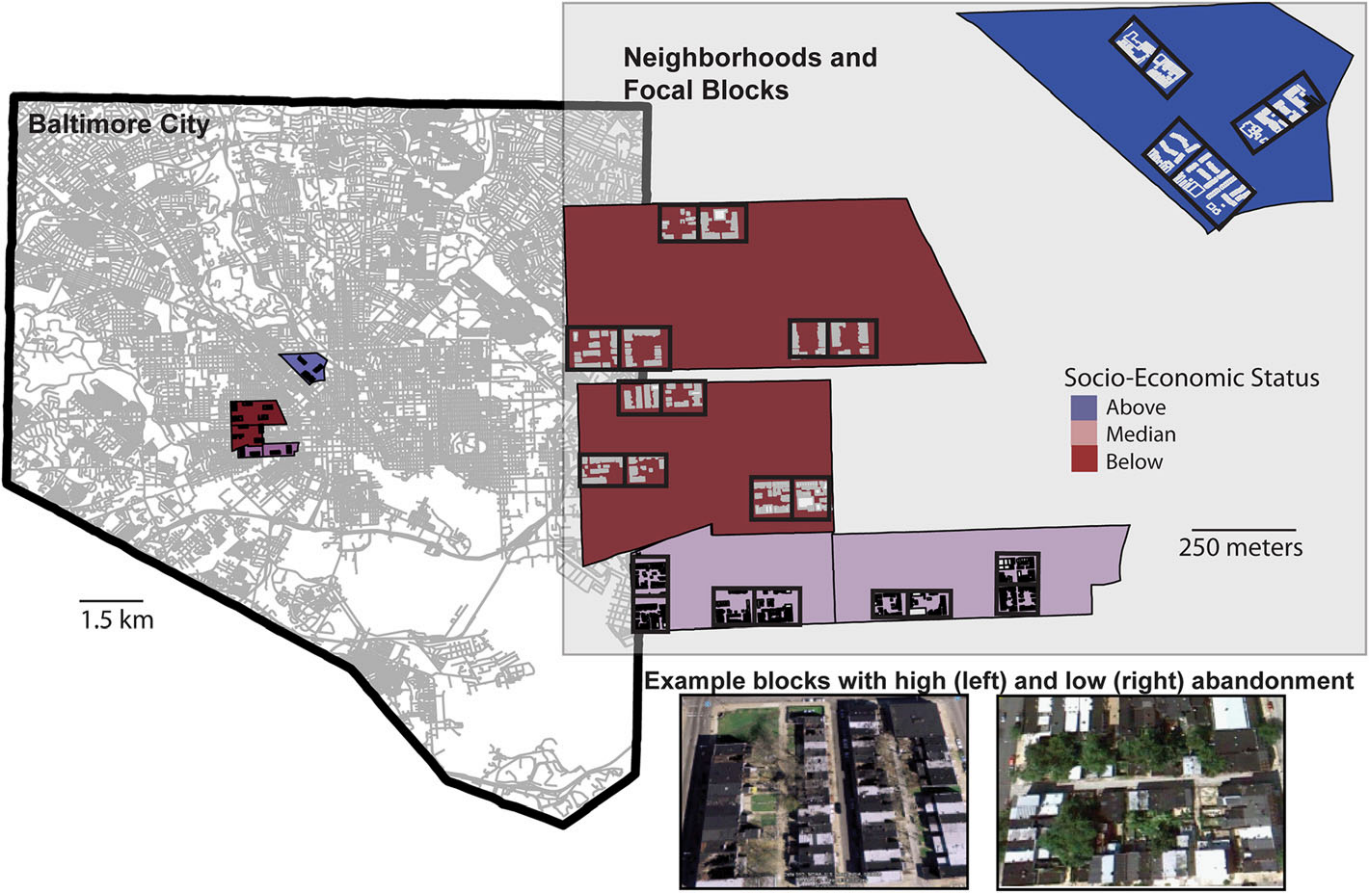
Map of study neighborhoods and focal blocks in Baltimore City, Maryland, USA. Aerial images from Google Maps (Imagery@Google 2016) shown depict examples from the focal study area of blocks with high and low infrastructure abandonment

We focused data collection in 5 neighborhoods that (i) consisted of row homes; (ii) were categorized as having household incomes (roughly $15,000) Below, Above or at (Median) the City’s median household income of $41,000 (in 2010); and (iii) were more than one kilometer from two large City Parks (Druid Hill and Leakin Park) and the Inner Harbor (Fig. 1). Neighborhoods were identified using online data from Baltimore City and the US Census Bureau (http://bniajfi.org/ and https://www.census.gov). This work was conducted as part of a larger coupled natural human systems project designed to investigate mosquito and human systems in the context of individual and community actions and urban decay (i.e. population decline, abandoned lots and unmanaged refuse) in Baltimore, Maryland. For further information about neighborhood selection and confirmation of socio-economic status (SES), see [60]. Each of the five neighborhood boundaries varies in total area but city blocks within neighborhoods are relatively consistent in size (Table 1). We sampled adult mosquitoes intensively on 2–3 blocks per neighborhood that were randomly selected from all blocks identified as predominantly residential (avoiding blocks with schools, large apartment complexes and businesses) and that were separated by at least one unsampled block (13 blocks total). Adults were only sampled on two blocks in the ‘Median’ category to maintain the minimum distance between blocks in these smaller neighborhoods. In addition to adult mosquito sampling, infrastructure condition metrics were recorded for each focal block, as described in [60].

**Table 1:** Neighborhood socio-economic status (SES), area, and sampling coverage

Neighborhood	SES	Median home price (2016 US$)	NBHD area (km^2^)	Mean block area (km^2^)	Blocks (2 traps per block)	Per trap area (m^2^)	Trap days (24-hour trap periods)	Mean occupancy (% parcels per block)
Harlem Park	L	16,500	0.808	0.026	3	12,888	168	0.457
Franklin Square	L	21,250	0.451	0.021	3	10,311	168	0.505
Hollins Market	M	67,000	0.277	0.018	2	9242	112	0.950
Union Square	M	115,310	0.168	0.019	2	9561	112	0.785
Bolton Hill	H	355,000	0.58	0.023	3	11,496	168	0.986

### Surveillance

BG-Sentinel^TM^ traps baited with CO_2_ and a 2.0 ml Octenol Lure (a mammal-derived attractant) were used to trap adult, blood-seeking mosquitoes in each study year. Traps were deployed at two locations on each focal block. While this trap was designed to target host-seeking *Aedes*, it has been demonstrated to effectively sample blooded specimens of both *Aedes* and *Culex* species when used with a BG lure [44]. A preliminary study in two of the focal neighborhoods collected greater numbers of both *Culex* and *Aedes* in the BG-Sentinel^TM^ traps than in paired CDC light traps, both baited with CO_2_ [64].

Trap sites were selected on each 0.5 block area to maximize distance between traps (50–100 m) and according to where researchers could establish property access. Trapping for 2015 and 2016 occurred on three blocks each in our ‘Above’ and ‘Below’ category SES neighborhoods and two each in the smaller ‘Median’ SES neighborhoods (Table 1). Traps were deployed and operational for 72 h every three weeks between May and October. After 24 h, batteries, dry ice and catch bags were replaced at each trap. Mosquito catch bags were transported on dry ice and placed at -20 °C until processed, which occurred within 2 weeks of each trap date. In 2015, the trapping cycle began May 26–27th and ended October 21–22nd, for a total of 8 trapping sessions. Trapping in 2016 started June 6–7th and ended October 11–12th, for a total of 7 trapping sessions. Trapping efforts were ended when the total number of mosquitoes collected across all sites was less than 25 individuals.

Female mosquitoes from each trap were sorted by genus and enumerated. Female *Aedes* and some *Culex* mosquitoes were morphologically identified to species [65], and checked for the presence of a blood meal under a dissecting microscope. *Culex pipiens* and *Cx. restuans* [66] were differentiated using a molecular assay during blood meal processing [67]. Each female mosquito with a visibly engorged abdomen was placed in a microcentrifuge tube with silica gel beads (Grade 48, 4–10 mesh size) and a small piece of cotton, and stored at room temperature [68, 69]. Desiccation was chosen as the storage protocol due to lack of access to an ultra-low temperature (-80 °C) freezer, although this may have limiting consequences on amplification success [70].

### Molecular processing and blood meal identification

Abdomens were removed from blood-fed *Culex* and *Aedes* mosquitoes by using forceps to apply pressure between the thorax and abdomen against the wall of an Eppendorf tube. Between dissections forceps were dipped in 70% ethanol and passed through a flame to prevent cross-contamination. DNA from the blood in the abdomens was extracted using a Qiagen DNeasy Blood and Tissue kit (Qiagen Sciences, Germantown, MD, USA). To identify the source of the blood meals, we followed a protocol that first distinguishes human from non-human mammal-derived blood meals using a multiplex PCR assay developed for use in *Ae. albopictus* [71]. Samples that did not amplify with this assay were subjected to additional rounds of PCR screening with specific avian [72], mammalian [73] and reptile/amphibian [74] primers. Negative controls (PCR master mix and sterile water) were used to test for contamination in all reactions. With the exception of the species-diagnostic human band produced by the multiplex assay [71], all PCR products were cleaned and prepared for sequencing. Single bands were cleaned with Exo-Sap-IT (USB Products, Cleveland, OH, USA). In the case of a human/non-human mammal mix displaying two bands, the non-human band was excised from the gel and cleaned with a QIAquick Gel Extraction Kit (Qiagen Sciences, Germantown, MD, USA). Purified PCR products were cycle-sequenced with the forward primer of each pair and run on capillary automated sequencers (GenScript, Piscataway, NJ, USA). Sequences were entered in a BLAST search (Basic Local Alignment Search Tool) in GenBank to identify known species matches (> 98% similarity) [68].

All statistical summaries and graphics were completed using the R Statistical Software package 3.3.1. We discuss differences in host-use and mosquito abundances across neighborhoods, making the assumption that there is modest exchange or dispersal between neighborhoods. This is based both on negative sampling results from roads between blocks and the correspondence of adult and juvenile abundances at the block scale [75]. We do not assume spatial independence between the two traps on a given block. Information from both traps on a block is combined in one per-trap-night estimate per sample period per block. Chi-square statistics were employed to assess significant differences in proportional host-use across across neighborhoods and SES category.

## Results

**Fig. 2:**
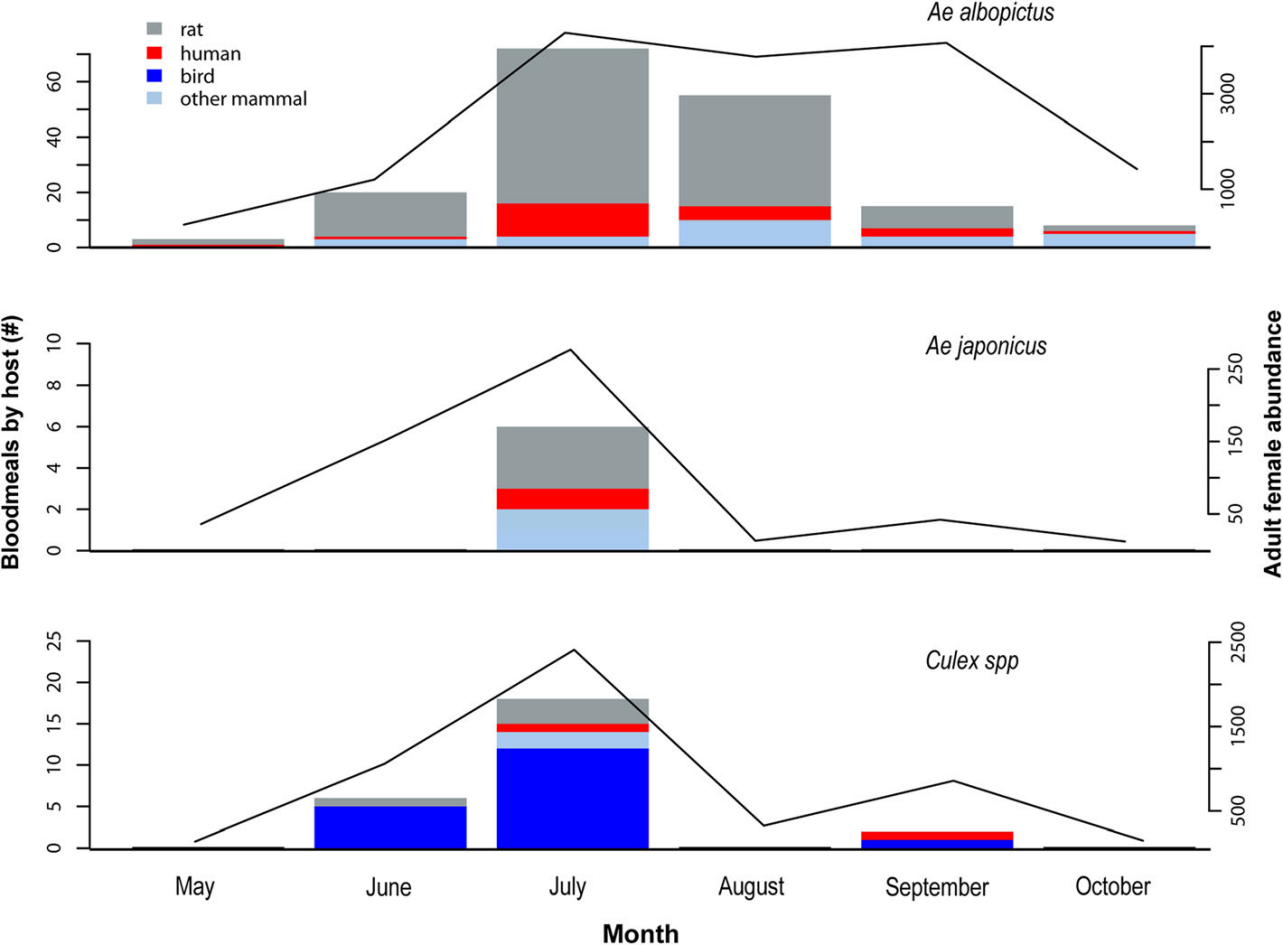
Bars show the number of host blood meals by species group detected in *Ae. albopictus*, *Ae. j. japonicus* and pooled *Culex* specimens in each month (left axis) and are overlaid with total number of female mosquitoes of each species (solid lines, right axis)

A total of 20,551 adult female mosquitoes were collected across both years (49% in 2016). *Aedes albopictus* made up 73.1% (*n* = 15,023) of the total collected mosquitoes, 24.1% (*n* = 4947) were *Culex* species, and 2.4% (*n* = 531) were *Ae. j. japonicus*. Blood-fed mosquitoes were collected at 23 of the 26 trap sites and from all focal blocks. Fifty (< 1%) additional specimens were identified across the two years as *Anopheles*, *Culiseta*, *Coquillettidia* and *Ae. aegypti*. The majority of blooded mosquitoes were collected in July (Fig. 2). Blood fed females were generally more frequently sampled when total abundance was greatest, as for *Culex* and *Ae. j. japonicus,* although numbers of blood-fed *Ae. albopictus* was low in September despite persistently large populations (Fig. 2). Only one specimen with blood meals from multiple species was detected. One *Ae albopictus* collected in October from the Union Square neighborhood contained both human and cat DNA.

There were 208 (1.4%) *Ae. albopictus* that were visibly engorged and we successfully extracted DNA and determined blood meal origin from 177 individuals (Table 2). The proportion engorged relative to total *Ae. albopictus* females was significantly lower in the ‘Above’ income neighborhoods (*χ*^2^ = 25.91, *df* = 2, *P* < 0.001) as compared to either the ‘Median’ or ‘Below’ category neighborhoods (0.20% compared to 1.8% and 1.4%, respectively). The proportion of engorged *Ae. j. japonicus* collected relative to total adult female *Ae. j. japonicus* ranged from 1.3–2.6% and was not significantly different across neighborhood SES (*χ*^2^ = 0.67, *df* = 2, *P* = 0.71). *Aedes j. japonicus* was only detected at our sites in July of either year. The brown rat (*Rattus norvegicus*) was the most frequently detected blood meal source in both *Aedes* species, making up 72% of all *Ae. albopictus* blood meals and 50% of *Ae. j. japonicus* host species (Table 2). Additional *Ae. albopictus* blood meals came from humans (14%), domestic cats (12%), dogs (1%) and white-tailed deer (< 1%). Additional *Ae. j. japonicus* hosts included an even split across human, white-tailed deer and cat (Table 2). There were no avian hosts detected in blood meals from either *Aedes* species.

**Table 2:** Percent host blood meals and number of sequences (*n*) for *Aedes* species. Other *Aedes albopictus* hosts includes 2 dog blood meals from Franklin Square and 1 white-tailed deer blood meal from Harlem Park. A deer blood meal was also identified from *Ae. j. japonicus* in Union Square

Neighborhood	SES	*Ae. albopictus*				*Ae. j. japonicus*			
		Human (*n*)	Rat (*n*)	Cat (*n*)	Other (*n*)	Human (*n*)	Rat (*n*)	Cat (*n*)	Other (*n*)
Franklin Square	L	7.5 (4)	71.6 (38)	17.0 (9)	3.8 (2)	0 (0)	66.7 (2)	33.3 (1)	0 (0)
Harlem Park	L	4.5 (2)	72.7 (32)	20.5 (9)	2.3 (1)	100 (1)	0 (0)	0 (0)	0 (0)
Hollins Market	M	31.3 (10)	56.3 (18)	12.5 (4)	0 (0)	0 (0)	0 (0)	0 (0)	0 (0)
Union Square	M	13.6 (6)	86.4 (38)	0 (0)	0 (0)	0 (0)	0 (0)	0 (0)	100 (1)
Bolton Hill	H	50.0 (2)	50.0 (2)	0 (0)	0 (0)	0 (0)	100 (1)	0 (0)	0 (0)
Total area		13.6 (24)	72.3 (128)	12.4 (22)	1.7 (3)	16.7 (1)	50.0 (3)	16.7 (1)	16.7 (1)

**Fig. 3:**
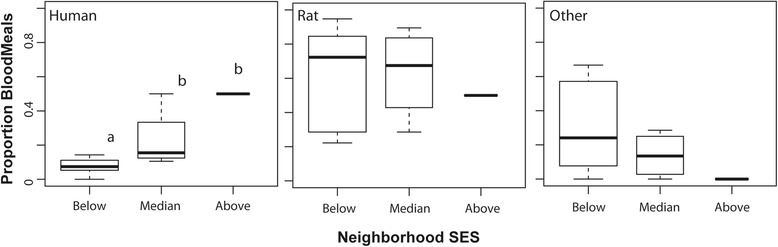
Boxplots show median and quartile values for relative proportions of human, rat, and other mammals (cat, dog and deer) hosts detected in *Ae. albopictus* from each SES category

There was a significantly lower proportion of *Ae. albopictus* blood meals taken from human hosts in the ‘Below’ category neighborhoods (*χ*^2^ = 12.63, *df* = 2, *P* = 0.002). Human blood made up the greatest proportion of total blood meals (50%) in ‘Above’ SES sites and was lowest (6%) in the ‘Below’ SES neighborhoods. Median income neighborhoods had significantly higher human blood meal proportions than the ‘Below’ income neighborhoods but were not significantly different from the ‘Above’ neighborhood samples (Fig. 3). SES category was not a significant predictor of relative proportions of *Ae. albopictus* blood meals from any of the non-human sources.

We visually identified 82 (1.7%) engorged *Culex* specimens and successfully determined blood meal origin for 26 individuals. The proportion engorged relative to total female *Culex* collected ranged from 1.2–2.5% and was not significantly different across neighborhood SES (*χ*^2^ = 3.17, *df* = 2, *P* = 0.204). Blooded *Culex* samples included three species: *Cx. pipiens* (65%), *Cx. restuans* (12%) and *Cx. salinarius* (23%). Avian comprised 100% and 94% of *Cx. restuans* and *Cx. pipiens* hosts, respectively, while *Cx. salinarus* hosts were entirely mammalian (Table 3). American robin (*Turdus migratorius*) was the most common avian blood meal source (44.4%) and these were collected predominantly from the ‘Below’ SES sites (6 of 8). Other avian hosts included the American crow (*Corvus brachyrhynchos*, *n* = 1), European starling (*Sturnus vulgaris*, *n* = 1), gray catbird (*Dumetella carolinensis*, *n* = 2), house sparrow (*Passer domesticus*, *n* = 1), mourning dove (*Zenaida macroura*, *n* = 4) and an unknown thrush (Turdidae, *n* = 1). Human blood was only detected in *Culex pipiens* samples (6%) both during peak abundance in July and again in September, when *Cx. pipiens* population abundance was relatively low (Fig. 2). There were no statistical differences in the relative proportions of human or non-human blood meals from *Culex* sampled across the SES categories.

**Table 3:** Percent blood meal and sequence number (*n*) for *Culex* species

Neighborhood	SES	*Cx. restuans*			*Cx. pipiens*			*Cx. salinarius*			
		Human (*n*)	Avian (*n*)	Rat (*n*)	Human (*n*)	Avian (*n*)	Rat (*n*)	Human (*n*)	Avian (*n*)	Rat (*n*)	Cat (*n*)
Franklin Square	L	0 (0)	100 (1)	0 (0)	12.5 (1)	87.5 (7)	0 (0)	0 (0)	0 (0)	0 (0)	0 (0)
Harlem Park	L	0 (0)	100 (1)	0 (0)	0 (0)	100 (3)	0 (0)	0 (0)	0 (0)	66.6 (2)	33.3 (1)
Hollins Market	M	0 (0)	0 (0)	0 (0)	0 (0)	100 (3)	0 (0)	0 (0)	0 (0)	0 (0)	0 (0)
Union Square	M	0 (0)	100 (1)	0 (0)	0 (0)	100 (1)	0 (0)	0 (0)	0 (0)	100 (1)	0 (0)
Bolton Hill	H	0 (0)	0 (0)	0 (0)	0 (0)	100 (2)	0 (0)	0 (0)	0 (0)	0 (0)	100 (1)
Total area		0 (0)	100 (3)	0 (0)	5.88 (1)	94.1(16)	0 (0)	0 (0)	0 (0)	60 (3)	40 (2)

## Discussion

This study highlights fine-scale variation in mammalian and avian host-use by medically important mosquito vectors and specifically investigates blood meal composition at spatial scales relevant to urban mosquito dispersal and human exposure. Further, the work emphasizes the importance of neighborhood socio-economic status and physical condition in shaping both the relative abundance of vectors and their local affinity for avian, mammalian and especially, human blood hosts.

The primary WNV vectors in the region, *Cx. pipens* and *Cx*. *restuans*, were predominantly ornithophagic, consistent with previous work [23, 44, 76]. The most frequently identified avian DNA was from the American robin, a known WNV reservoir and amplification species [23, 27]. However, while American robin blood was detected in specimens from all five neighborhoods, human blood meals were only found in *Cx. pipiens* collected from ‘Below’ SES sites. *Culex* species tend to bite at dawn and dusk, unlike the daytime biting *Aedes*, and it is not clear whether this reflects differences in dawn and dusk access to humans across neighborhoods or is just a stochastic result of small sample size. The small number of engorged *Culex* specimens (0.5% of total females collected) may indicate need for amending sampling and processing protocols to either attract more blooded *Culex* or better preserve the collections prior to PCR. Resting traps were trialed in our study sites but yielded no blooded *Culex*. *Culex* species may disperse several kilometers from juvenile breeding sites to find blood meal hosts. While we do find all three adult *Culex* species in our juvenile sampling across these same neighborhoods [29], it is likely that there is movement between neighborhoods to locate preferred hosts. While not the focus of the current study, future work will examine host-use of different avian species in the context of variable avian composition and abundance across different neighborhood SES conditions.

This work is consistent with previous studies that report opportunistic but mammalian-focused feeding strategies for *Ae. albopictus* and *Ae. j. japonicus*. However, we further demonstrate that host feeding by *Ae. albopictus* in particular differs significantly across SES categories. *Aedes albopictus* dispersal is generally less than 100 meters, or the distance across one of our city blocks [46, 77]. Thus, our results indicate that a female *Ae. albopictus* that developed on a higher income block is more likely to take a human blood meal than a mosquito emerging on a lower income block. However, vectorial capacity (and thus, transmission risk) is also a function of vector abundance [7, 13]. Female *Ae. albopictus* abundance has previously been associated with SES category in these same neighborhoods - with greatest abundance on lower income blocks that have high infrastructure abandonment [29, 60]. The greatest capacity for vector transmission of pathogens among humans occurs where both vector abundance and human host-use are maximized. In this system these conditions are both maximized in the median income neighborhoods where *Ae. albopictus* abundance is greater than in neighborhoods with higher SES and human blood meal proportion is higher than in the lower income neighborhoods (e.g. Fig. 3). We believe that the greater proportion of abandoned infrastructure in the lower income neighborhoods, where proportion of buildings occupied ranged from 23–54% (mean 37%) across individual blocks, indicates a loss of access to human blood meals that is further reduced because remaining residents are less likely to spend time in backyards or shared green space (LaDeau, unpublished data). By comparison, a greater proportion of homes in the ‘Median’ category neighborhoods were occupied 69–96% (mean 83.5%) and residents were generally more likely to use local community gardens and shared green spaces (LaDeau, unpublished data). This study suggests that the relevant parameters necessary to understand vectorial capacity and both the rate and frequency of arboviral transmission in the urban landscape vary significantly at fine scales within and between urban neighborhoods. Perhaps more importantly, the current study indicates that knowing something about local infrastructure management and resident behavior may help guide management strategies and generate testable hypotheses for where transmission is most likely.

The invasive brown rat (also called Norway rat) is an important blood meal source across vector species and neighborhoods in Baltimore. Brown rats are a globally ubiquitous rodent in urban ecosystems, inhabiting sewer systems and dirt burrows. Urban rats have short dispersal distances and population structure is evident even at the scale of a city block [78]. Although most rat activity occurs just before or at sunset when *Culex* species feed, younger rats forage earlier in the day to avoid competing with larger, dominant rats [79]. This may make them more susceptible to being bitten by *Aedes*, as both *Ae. albopictus* and *Ae. j. japonicus* are daytime feeders. In 2004, researchers described a large, persistent brown rat population in Baltimore, MD, and trapped a majority of rats from alleys categorized as being in lower income neighborhoods [80]. Nearly 50% of the trapped rats across all sites tested positive for Seoul virus, a hantavirus that has also been detected locally in humans [81]. Rats are known reservoirs for other pathogens as well, including *Salmonella*, *Leptospira*, *Rickettsia* and *Bartonella* [82–86] and may be effective reservoirs for some arboviruses [87–89].

Samples from below SES neighborhoods included both a higher proportion and greater number of rat blood meals detected. This is consistent with being where the rat populations are believed to be most abundant [80]. These lower income neighborhoods are visibly characterized by infrastructure abandonment. Abandonment is evident in boarded doors and windows and in some cases, missing roofs (Fig. 1), but also because of the unmanaged refuse that is often illegally dumped by people external to the neighborhood. Previous work by this group has shown that these conditions support high numbers of unmanaged containers that are important habitat for immature development and production of adult *Culex* and *Aedes* populations [29, 60]. The majority of cat blood meals also were found in these lower income sites (Table 2), where feral cats have been fed and even encouraged in response to the rat problem. The current study raises important questions about the role of rat populations in supporting urban mosquito populations, the possibility of rat-prophylaxis (diversion of bites from humans) as well as their potential to act as pathogen reservoirs. It is unknown, for instance, whether a mosquito that bites a rat is more likely to feed to repletion before being disturbed or have different fitness consequences than a mosquito that bites a human. Understanding the mechanistic role that rodents play in feeding urban mosquito populations is important for predicting how pest management interventions and changes in infrastructure might influence mosquito abundances and human biting pressure.

Although *Ae. albopictus* has been endemic in the Baltimore region for nearly three decades, *Ae. j. japonicus* was only detected in Maryland in 2000. This relative newcomer, was less abundant across all focal neighborhoods and only six engorged females were sampled over the two years of the study. Despite the low number of blood-fed *Ae. j. japonicus* examined, the blood sources identified included the range of hosts seen in the more abundant and widespread *Ae. albopictus*. We identified one human blood meal in *Ae. j. japonicus* and though detection of human feeding in this species is not new [58], the opportunistic nature of its blood feeding behavior implicates it as a potentially important vector for La Crosse virus or other zoonoses with small mammal reservoirs [2, 56, 90].

The socio-economic indicators of arboviral transmission risk are complex and can covary with environmental drivers, such as temperature and precipitation [60, 64, 91, 92]. Furthermore, while higher SES neighborhoods often have lower human population densities due to larger residential footprints, urban population loss across many temperate cities is often focused in lower income neighborhoods [63]. Abundant mosquito vectors developing in the abandoned infrastructure across these neighborhoods may be less likely to encounter a human at all much less twice, as would be needed to first acquire and then transmit a non-zoonotic virus. Furthermore, this group has found that the residents in lower income Baltimore neighborhoods were more likely to spend time in the front of the row homes, where streets are paved and there is little shade or water-holding habitat for mosquitoes. By contrast, residents in higher income neighborhoods were more likely to maintain personal recreation space behind the home and to report perceived mosquito nuisance while using these spaces [29, 75]. Assessing vectorial capacity in these complex urban landscapes requires some fine-scale geographical understanding of the biophysical and socio-economic conditions and human behaviors that influence juvenile and adult mosquito life stages.

## Conclusions

This study demonstrates how fine-scale variation in host-use by medically important mosquito vectors can define a gradient in human exposure to mosquitoes and associated arboviruses across city neighborhoods. Further, the work emphasizes the importance of neighborhood socio-economic status and infrastructure management in shaping both the relative abundance of vectors and local affinity for avian, mammalian and especially, human blood meals. The greatest capacity for vector transmission of pathogens among humans occurs where both vector abundance and human host-use are maximized. In Baltimore this is in the median income neighborhoods where heterogeneous land management supports higher mosquito abundance than in high SES neighborhoods but resident use of outdoor spaces likely supports higher proportion human blood hosts than in low income neighborhoods. The invasive brown rat is an important blood source across vector species and sites, although it is a most frequent blood host in lower income neighborhoods. The current study raises important questions about how variation in rodent abundance and access to humans across the urban landscape can influence mosquito population growth, human biting pressure and potential transmission of endemic and emergent arboviruses.
